# Supplementary material for: Improvement of Dynamic Performance and Detectivity in Near-Infrared Colloidal Quantum Dot Photodetectors by Incorporating Conjugated Polymers
Source: Molecules. 2022 Nov 7;27(21):7660. doi: 10.3390/molecules27217660 (PMC9655137; doi:10.3390/molecules27217660)
Supplement: Supplementary file 1 [file molecules-27-07660-s001.zip › molecules-1938176-supplementary.pdf]

Supporting Information

Improvement of Dynamic Performance and Detectivity in Near-Infrared Colloidal Quantum Dot Photodetectors by Incorporating Conjugated Polymers

Myeong In Kim [1], Jinhyeon Kang [1], Jaehee Park [1], WonJo Jeong [1], Junho Kim [2], Sanggyu Yim [3,*] and In Hwan Jung [1,*]

[1] *Department of Organic and Nano Engineering, and Human-Tech Convergence Program, Hanyang University, 222 Wangsimni-ro, Seongdong-gu, Seoul 04763, Republic of Korea*

[2] *Department of Energy Engineering, Hanyang University, 222 Wangsimni-ro, Seongdong-gu, Seoul 04763, Republic of Korea*

[3] *Department of Chemistry, Kookmin University, 77 Jeongneung-ro, Seongbuk-gu, Seoul 02707, Republic of Korea*

*Correspondence: sgyim@kookmin.ac.kr (S. Yim); inhjung@hanyang.ac.kr (I. H. Jung)

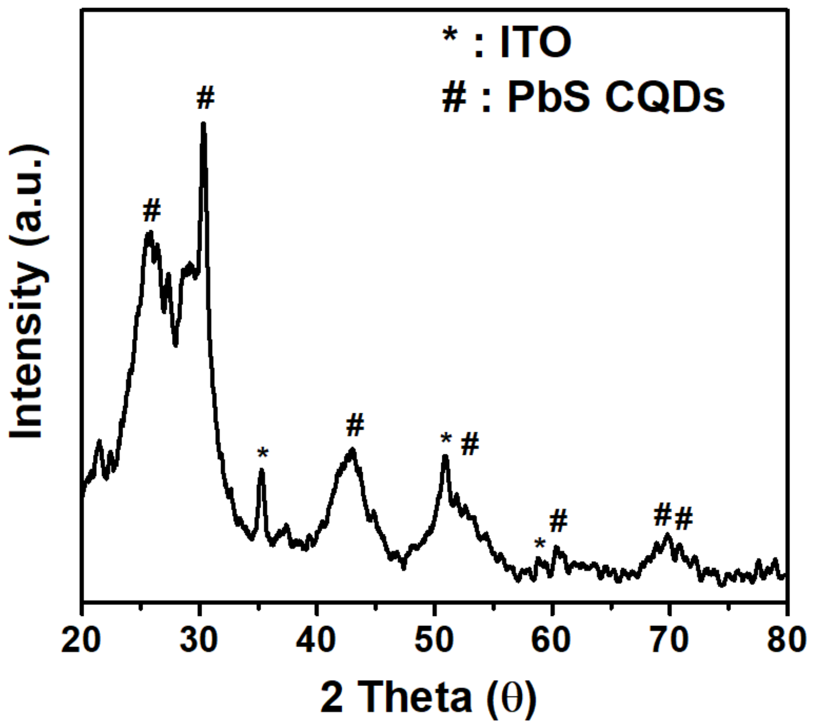

Figure S1. X-ray diffraction spectrum of PbS CQD coated on ITO-patterned glass.

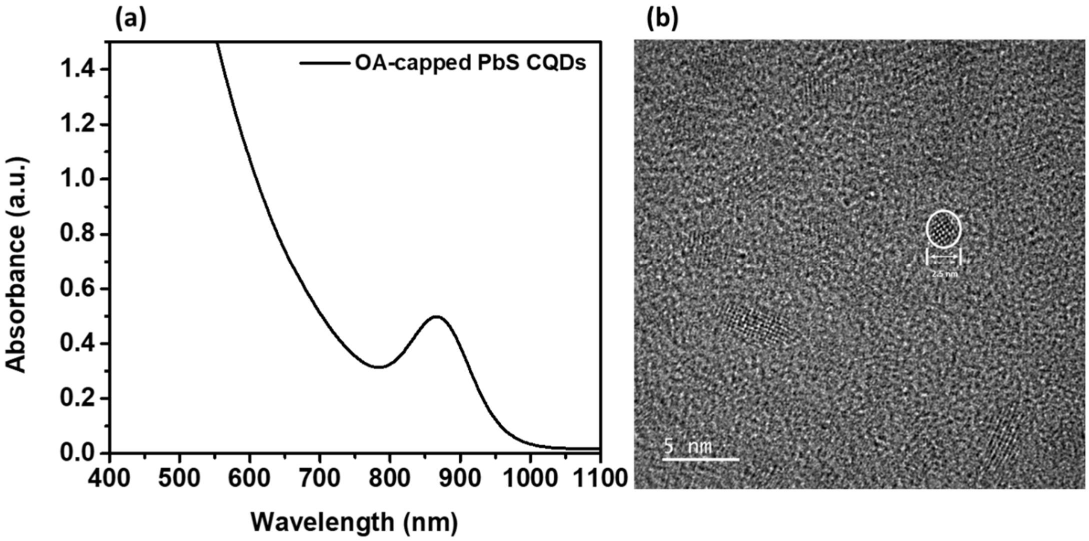

Figure S2. (a) UV/Vis absorption spectra and (b) TEM image of synthesized OA-capped PbS CQDs.

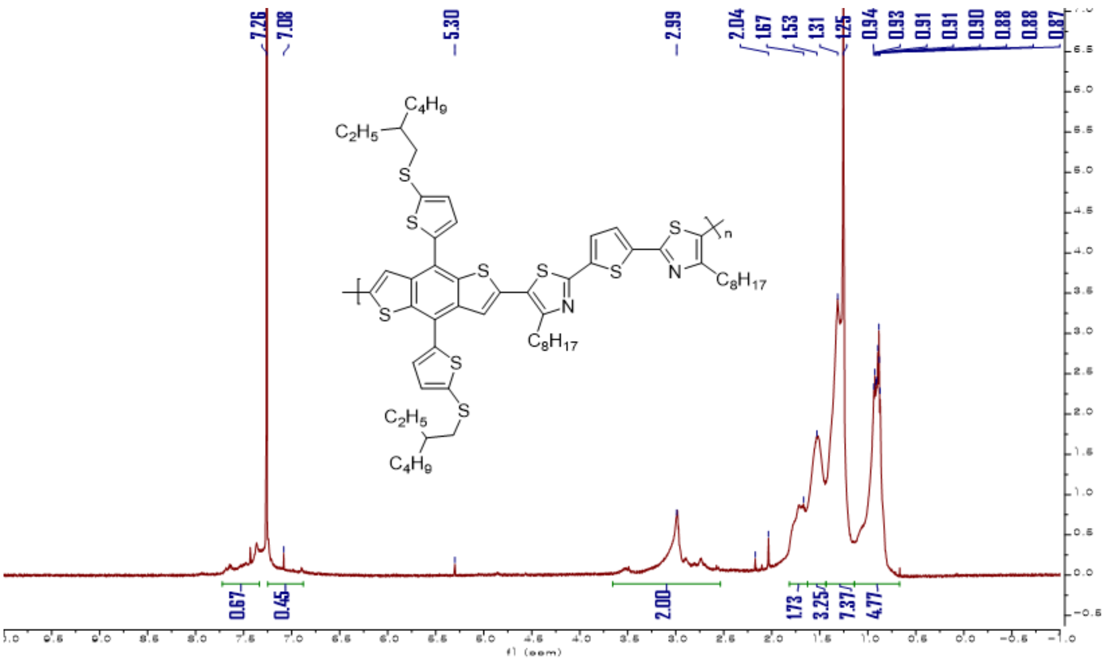

Figure S3. ^{1}H NMR of PSBOTz

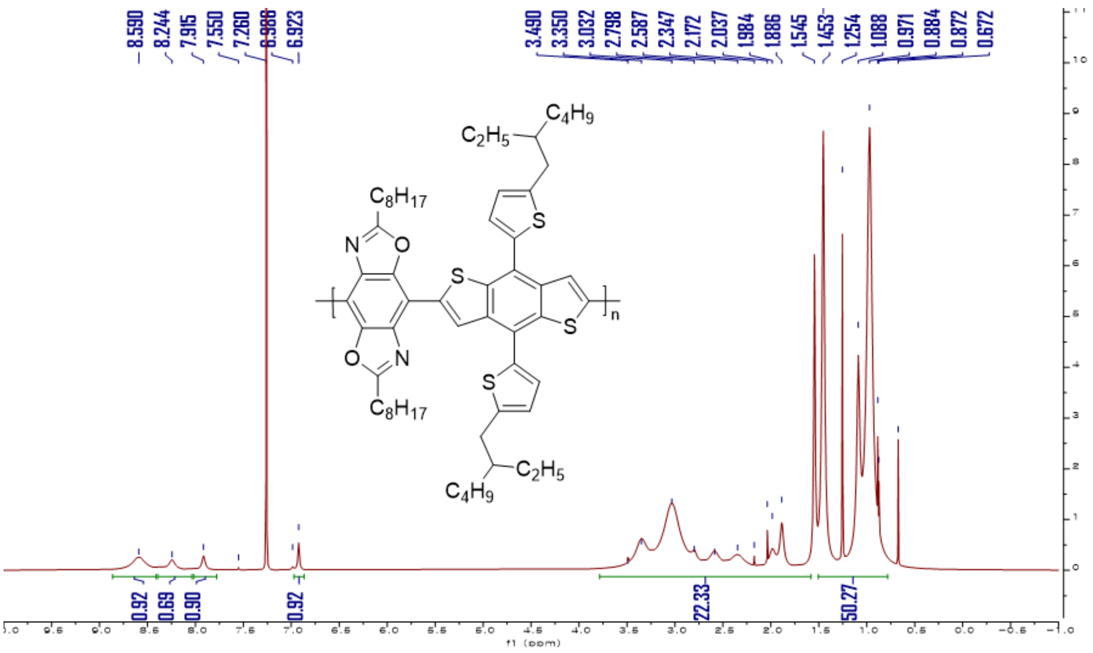

Figure S4. ^{1}H NMR of PBB

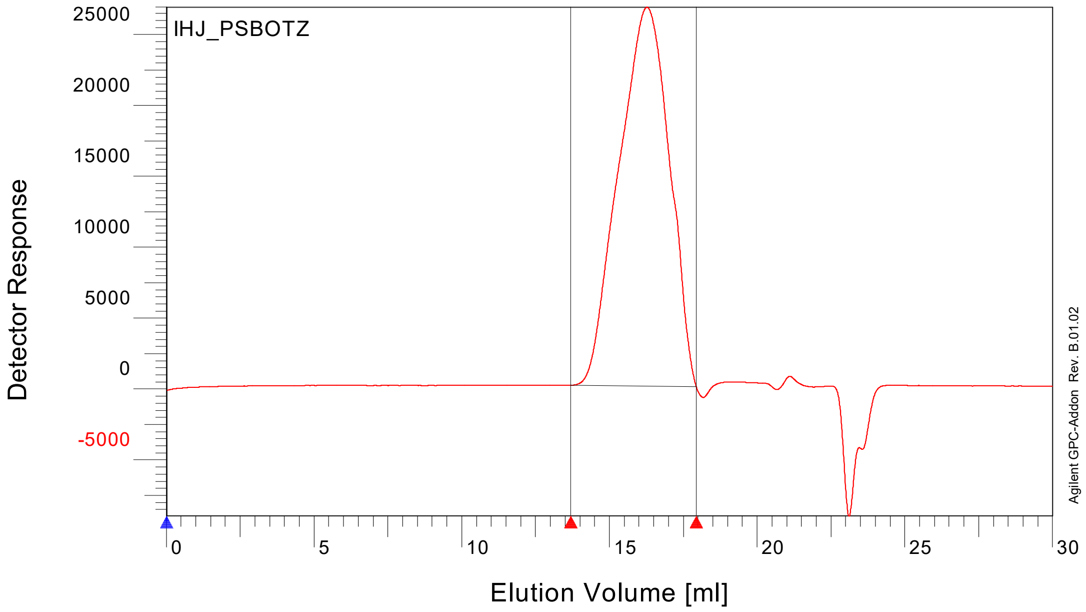

Figure S5. GPC results for PSBOTz.

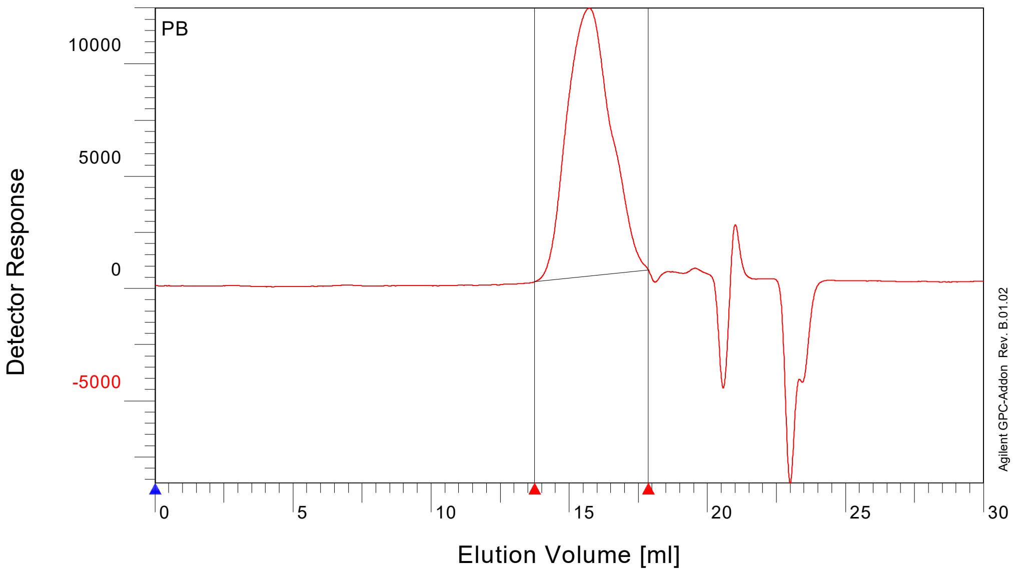

Figure S6. GPC results for PBB.

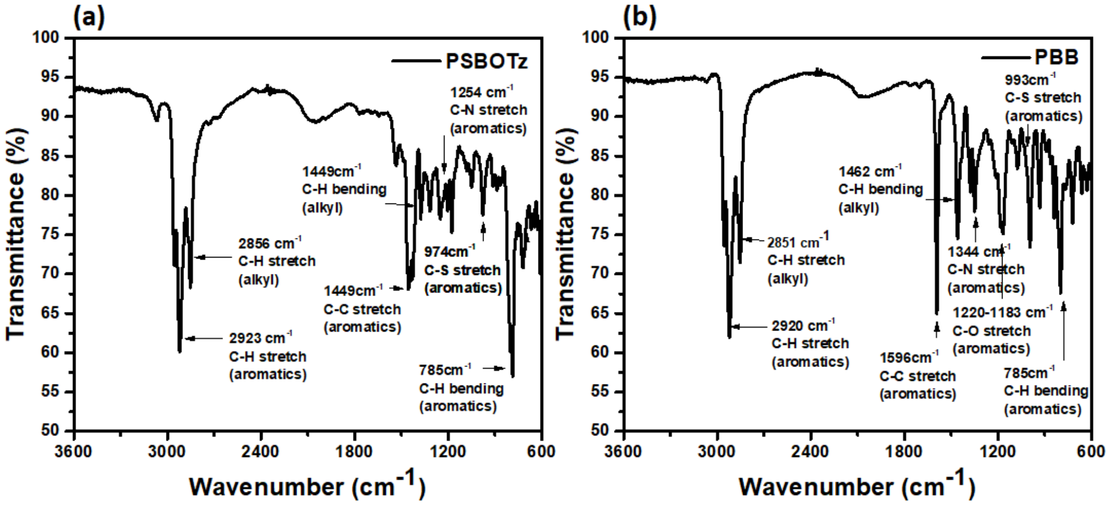

Figure S7. FT-IR spectra of (a) PSBOTz and (b) PBB conjugated polymers.

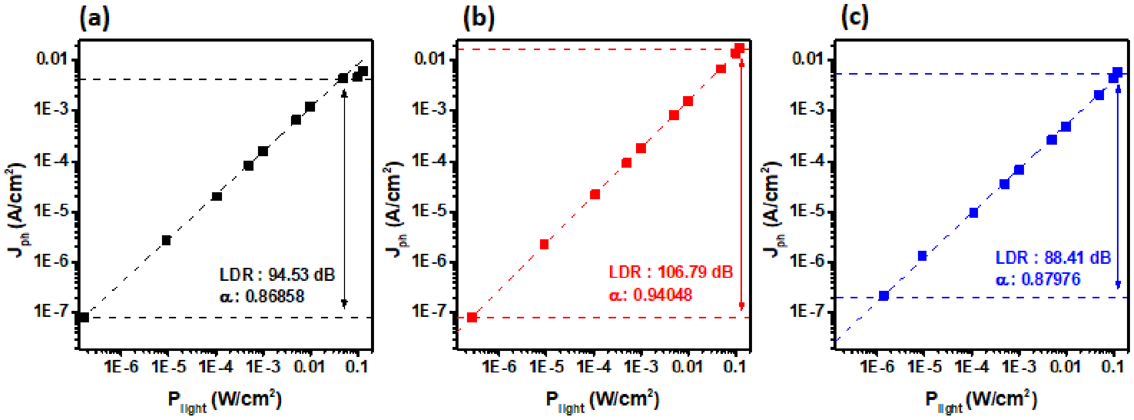

Figure S8. Linear dynamic range results for (a) PSBOTz, (b) PBB, and (c) PbS-EDT based devices at −1.0 V bias.

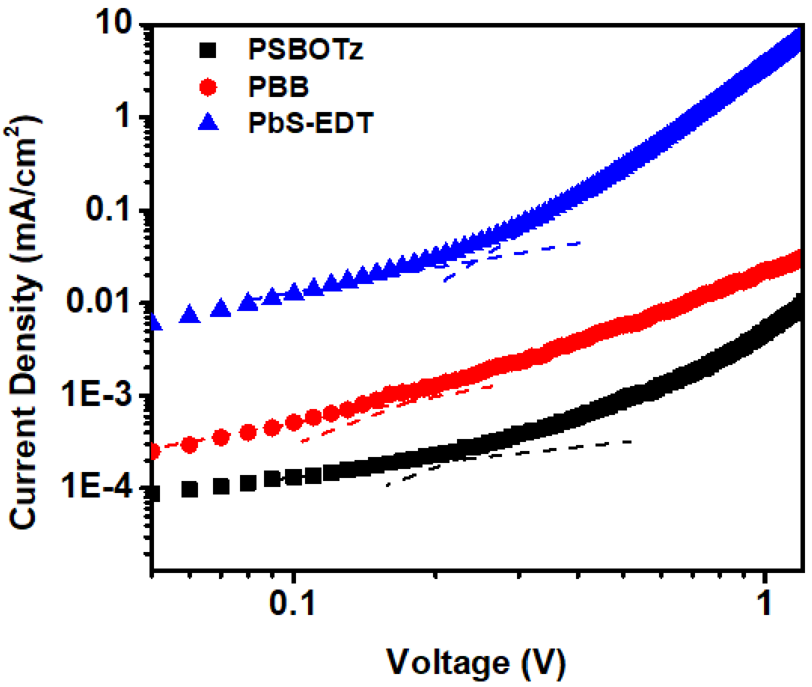

Figure S9. SCLC plots using an electron-only device (ITO/ZnO/PbS-EDT or CP/Al) for the trap density calculation.

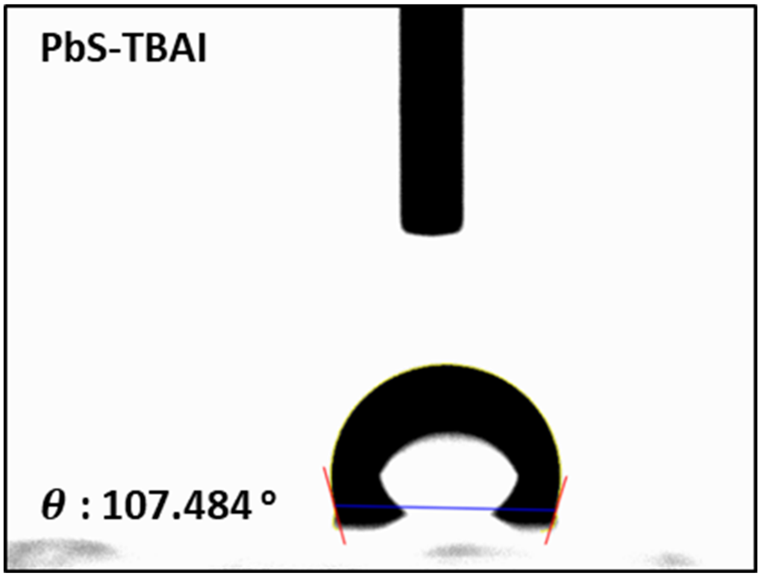

Figure S10. Water contact angle result for PbS-TBAI pristine film.

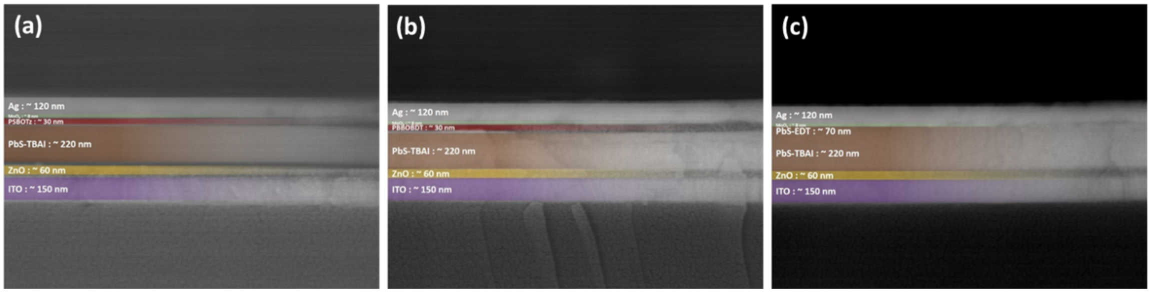

Figure S11. Cross-sectional SEM images of (a) PSBOTz, (b) PBB, and (c) PbS-EDT based devices.

Table S1. Summary of optical and electrochemical properties of the CPs in this study.

Polymer	λ_{max} [nm]		λ_{onset} [nm]	E_g^{opt} (eV)	HOMO (eV)	LUMO (eV)	E_g (eV)
	Solution	Film	Film				
PSBOTz	508	520	611.2	2.029	−5.054	−2.928	2.126
PBB	476	524	620.28	1.999	-−5.375	−3.364	

Table S2. Summary of calculated hole mobility, maximum charge generation rate, and trap density value of each fabricated device.

Hole Transporting Layer	Hole Mobility (cm^2 V^{-1} s^{-1})	Maximum Charge Generation Rate ($m^{-3}s^{-1}$)	Trap Density (cm^{-3})
PSBOTz	2.75×10^{-3}	1.13×10^{26}	9.487×10^{16}
PBB	9.63×10^{-3}	3.66×10^{26}	8.951×10^{16}
PbS-EDT	1.77×10^{-3}	8.36×10^{25}	1.276×10^{17}
